# Impact of Multifidus Muscle Atrophy on the Occurrence of Secondary Symptomatic Adjacent Osteoporotic Vertebral Compression Fractures

**DOI:** 10.1007/s00223-021-00925-1

**Published:** 2021-10-15

**Authors:** Georg Osterhoff, Garnik Asatryan, Ulrich J. A. Spiegl, Christian Pfeifle, Jan-Sven Jarvers, Christoph-E. Heyde

**Affiliations:** Department of Orthopaedics, Trauma and Plastic Surgery, University Hospital Leipzig, 04103 Leipzig, Germany

**Keywords:** Vertebral compression fracture, Spinal fracture, Kyphoplasty, Adjacent-level vertebral fracture, Adjacent vertebral fracture, Osteoporosis, Risk factor, Sarcopenia, Muscle

## Abstract

To assess the potential influence of multifidus atrophy and fatty degeneration on the incidence of adjacent vertebral compression fractures within one year after the index fracture. In a retrospective cohort study, patients who underwent surgery for an OVCF were identified and baseline characteristics, fracture patterns and the occurrence of secondary adjacent fractures within one year were obtained by chart review. Multifidus muscle atrophy and fatty degeneration were determined on preoperative MRI or CT scans. In this analysis of 191 patients (mean age 77 years, SD 8, 116 female), OF type 3 was the most common type of OVCF (49.2%). Symptomatic adjacent OVCFs within one year after index fracture were observed in 23/191 patients (12%) at mean 12, SD 12 weeks (range 1–42 weeks) postoperatively. The mean multifidus muscle area was 264, SD 53 mm^2^ in patients with an adjacent vertebral fracture and 271, SD 92 mm^2^ in patients without a secondary fracture (p = 0.755). Mean multifidus fatty infiltration was graded Goutallier 2.2, SD 0.6 in patients with an adjacent fracture and Goutallier 2.2, SD 0.7 in patients without an adjacent fracture (p = 0.694). Pre-existing medication with corticosteroids was associated with the occurrence of an adjacent fracture (p = 0.006). Multifidus area and multifidus fatty infiltration had no significant effect on the occurrence of adjacent vertebral fractures within one year after the index fracture. Patients with a pre-existing medication with corticosteroids were more likely to sustain an adjacent fracture.

## Introduction

Osteoporotic vertebral compression fractures (OVCF) are common fractures in the elderly—especially in postmenopausal women [1]. OVCF are frequently associated with severe immobilizing pain. This results in an increased morbidity and mortality of these bed-ridden patients. About 20% of the patients with a primary OVCF suffer subsequent fractures in the spinal segments adjacent to and within one year after the index fracture [2–4]

There is debate about what factors contribute to an increased risk for such adjacent fractures. Factors discussed so far include bone quality, age, gender, medical comorbidities, number of initial fractured levels, and fracture location [3–6].

Thoraco-lumbar core muscles have a relevant impact on the biomechanics of the spine and most patients with OVCF present with sarcopenia [7]. A recent study reported that in patients with OVCF, fatty degeneration of the paraspinal muscles is a predictive factor for progressive vertebral body collapse [8]. It is known that a decreased hand-grip strength is associated with an increased subsequent vertebral fracture risk [9]. The potential role of the autochthonous muscles of the back on the occurrence of secondary adjacent fractures, however, has—to the authors’ knowledge—not yet been evaluated.

The purpose of this retrospective study was therefore to assess the potential influence of multifidus atrophy and fatty degeneration on the incidence of adjacent vertebral compression fractures within one year after the index fracture—and to confirm other risk factors known from the literature.

It was our hypothesis that an impaired multifidus muscle support increases the likelihood for an adjacent fracture.

## Materials and Methods

A monocentric retrospective cohort study was conducted at a university level 1 trauma center through review of charts. This study was approved by the local institutional ethics committee (reference 316/19-ek). The study was conducted in accordance with all local regulations and followed the principles of the Helsinki declaration. Informed consent was not obtained due to the retrospective nature of the study in accordance with §34 of the Saxon Hospital Act. Patients who earlier had expressed objection to the use of their data for research purposes were excluded.

### Patients

**Fig. 1:**
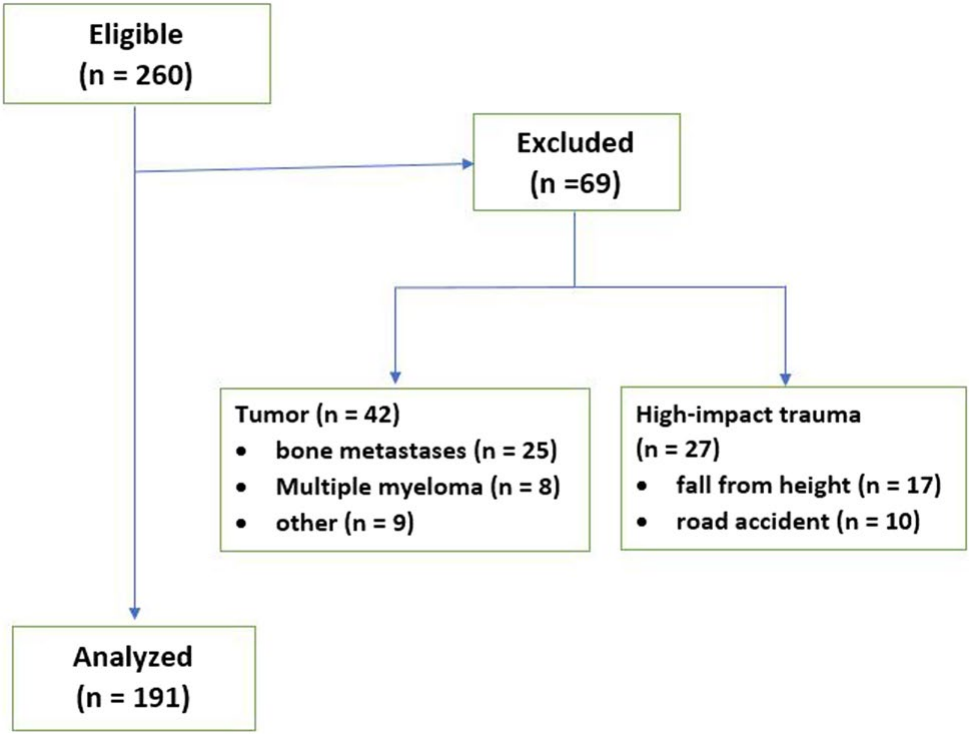
Patient flow chart

All consecutive patients aged 60 years and older who were treated operatively for an isolated thoraco-lumbar OVCF (T5 to L5) without neurologic deficits from January 2014 to December 2018 were identified. An OVCF was defined as having occurred spontaneously or as a result of minimal trauma from day-to-day activities [10]. Patients who had MRI and/or CT imaging of the fracture including axial reconstructions of the multifidus muscles within 4 weeks around the index fracture were included. Patients with high-energy trauma, B- or C-type injuries according to the AOSpine classification [11], or metastatic fractures were excluded (Fig. 1).

Indications for surgery were painful acute vertebral compression fractures (OF classification type 2, 3, 4, and type 1 if not responding to conservative treatment) [12]. Patients were treated by either kyphoplasty (OF type 2) or by percutaneous or open reduction and posterior fixation (OF 3 and 4) with pedicle screw-rod internal fixation (*USS II*, Synthes, Oberdorf, Switzerland or *Longitude*/*Legacy*, Medtronic, Fridley, MN, USA).

Patients in both groups received a treatment with calcium and vitamin D and—if this had not already been installed—further assessment and treatment was initiated according to the guidelines of the Austrian, Swiss, and German Umbrella Organization for Osteology (DVO, www.dv-osteologie.org).

Clinical routine follow-ups were performed at 12 weeks, 6 months, and 12 months, new imaging was performed only in case of complaints.

### Data Acquisition

Epidemiologic data, information on fracture, and treatment characteristics were obtained by chart review. Information on comorbidities was summarized in terms of the American Society of Anesthesiologists Scale (ASA).

**Fig. 2:**
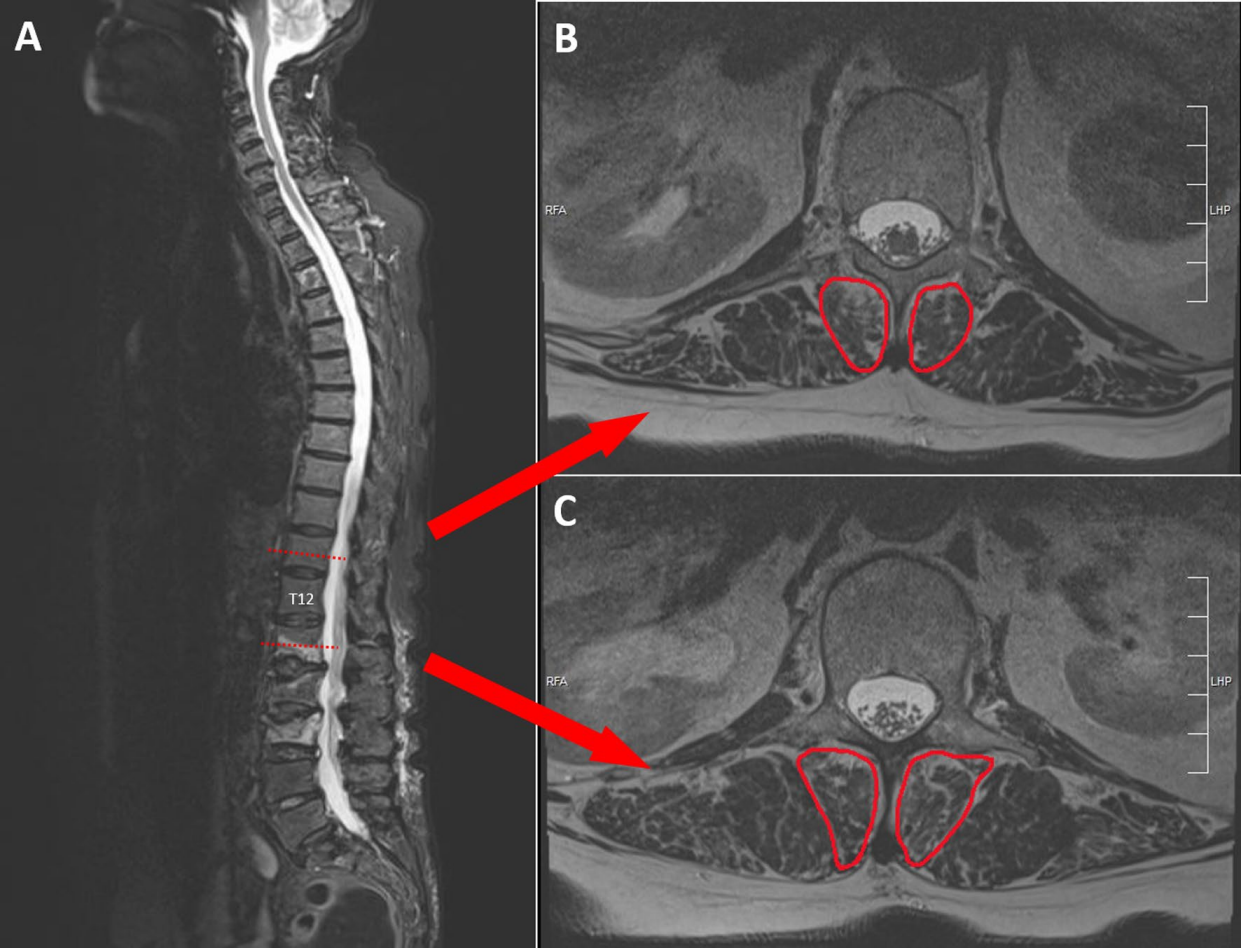
Measurement of cross-sectional area of the multifidus muscles

Multifidus muscle atrophy was determined by measuring the muscles’ cross-section area in MR imaging using an imaging processing software (ImageJ, version 1.8.0) [13]. Area measurements were performed bilaterally on axial reconstructions of the spine as described previously at two levels above and below T12 [14] and mean values of the four measurements per patient were calculated for analysis (Fig. 2).

On the same images, multifidus muscle fatty degeneration was graded by the Goutallier classification that was originally described for rotator cuff fatty infiltration [15]. Fatty infiltration of the muscle is described as normal (grade 0), containing some fatty streaks (grade 1), relevant fatty infiltration but more muscle than fat (grade 2), equal amounts of fat and muscle (grade 3), and more fat than muscle (grade 4). Measurements were performed at two levels and mean values were calculated for analysis.

In those patients, who had CT imaging of the thoraco-lumbar spine during the hospitalization, bone quality was assessed by measuring Hounsfield units in the adjacent vertebra [16]. Measurements were performed at three levels of the vertebral body just below the fractured vertebra and mean values were calculated for analysis (except for fractures of L5 where L4 was used for the measurements).

### Outcome

The primary outcome was the occurrence of a symptomatic adjacent osteoporotic vertebral fracture within one year after the index fracture that lead to a presentation in our department. All patients included had a follow of at least 12 months. An adjacent osteoporotic vertebral fracture was defined as a radiographically proven symptomatic fracture within two levels of the index vertebra.

### Statistical Analysis

A sample size calculation was performed prior to the investigation. Given a desired type 1 error of 0.05, a number of two predictors in the model (1. multifidus muscle area and 2. fatty degeneration), an anticipated medium effect size of 0.15, and a desired statistical power level of 0.80 this revealed a minimum required sample size of 67 [17].

All data were recorded in an Excel database (Microsoft Corp., Washington, DC, USA) and exported to SPSS 24.0 (SPSS Inc., Chicago, IL, USA) for statistical analysis. Continuous data were reported as mean with standard deviation (SD), categorical data as frequency (n), and percentage (%).

Primary outcome was the occurrence of an adjacent osteoporotic vertebral fracture within one year after the index fracture. A regression analysis was performed to assess “multifidus muscle area” and “multifidus muscle fatty degeneration” as potential independent risk factors for adjacent fractures.

To detect potential confounders like age, gender, fracture pattern (OF classification), comorbidity (ASA scale), type of surgery, bony quality (Hounsfield units) medication with bisphosphonates, vitamin D, or prednisolone crosstabs were associated. Where applicable, nominal variables crosstabs were associated using chi-Square or Fisher’s Exact tests. After testing for normal distribution, an independent Student’s t-test was used to compare continuous data. Multiple regression analysis was performed while adjusting for confounding factors that had been identified. The level of significance was defined as p < 0.05.

The intra-class correlation coefficient (ICC) and 95% CIs (two-way mixed, average measures, absolute agreement) were calculated to assess inter-observer agreement with regard to “multifidus muscle area”.

## Results

In total, 191 patients (mean age 77 years, SD 8, 116 female) were included into final analysis (Table 1). All patients had vertebral compression fractures, most of them OF type 3 (49.2%), followed by OF type 2 (30.9%), OF type 4 (19.4%), and OF type 1 (0.5%). For their index fracture, treatment consisted of kyphoplasty in 53 cases (27.7%), percutaneous posterior fixation in 95 (49.7%), and open fixation in 43 cases (22.5%). The mean ASA scale was 2.7, SD 0.6 with 124/191 (64.9%) presenting with an ASA of 3 or more.

**Table 1:** Patients’ baseline characteristics

	Adjacent vertebral fracture		p
	Yes	No	
N	23	168	
Age [y (SD)]	77 (7)	77 (8)	0.821^†^
Gender [f: m]	11: 15	86: 132	0.778*
ASA	2.8 (0.5)	2.6 (0.6)	0.224^†^
OF	2.7 (0.8)	2.9 (0.7)	0.211*
Medication			
Bisphosphonates—preop	1 (4.3%)	5 (3.0%)	0.542*
Bisphosphonates—postop	3 (13.0%)	11 (6.5%)	0.228*
Vitamin D—preop	6 (26.1%)	26 (15.5%)	0.162*
Vitamin D—postop	12 (52.2%)	68 (40.5%)	0.200*
Corticosteroids	6 (26.1%)	10 (6.0%)	0.006*
Bone quality (HU)	85 (45)	81 (35)	0.702^†^

*y* years, *SD* standard deviation, *f* female, m male, *ASA* American Society of Anesthesiologists Scale, *OF* osteoporotic fracture classification, *HU* Hounsfield units

*Pearson chi-square/Fisher’s exact test

^†^Student’s t-test

Adjacent vertebral compression fractures within one year after the index fracture were observed in 23/191 patients (12%). The mean time interval between surgery of the index fracture and diagnosis of a secondary adjacent fracture was 12, SD 12 weeks (range 1–42 weeks).

### Medication

Six patients (3.1%) had a medication with bisphosphonates before surgery and 14 patients (7.3%) at the time of discharge. A substitution with vitamin D had been established in 32 patients (16.8%) before surgery and was given in 80 patients (41.9%) after at the time of discharge. Sixteen patients (8.4%) were under medication with corticosteroids at the time of the index fracture. Medication—whether before or after the index surgery—with bisphosphonates and vitamin D was equally distributed between patients with and without an adjacent vertebral fracture within one year (p > 0.05, Table 1). Patients with an adjacent fracture, however, were significantly more likely to have pre-existing medication with corticosteroids (p = 0.006).

Bone quality as measured by Hounsfield units seemed not to have an impact on the occurrence of adjacent fractures in this cohort (p = 0.702).

### Multifidus Muscle Atrophy and Fatty Degeneration

The mean multifidus muscle area was 264, SD 53 mm^2^ in patients with an adjacent vertebral fracture and 271, SD 92 mm^2^ in patients without a secondary fracture (p = 0.755).

Intra-class correlation for the repeated measurements of multifidus muscle area was excellent with an ICC of 0.963 (95% CI 0.953–0.971, Cronbach’s alpha 0.963).

Mean multifidus fatty infiltration area was graded Goutallier 2.2, SD 0.6 in patients with an adjacent fracture and Goutallier 2.2, SD 0.7 in patients without an adjacent fracture (p = 0.694).

As corticosteroids revealed to be associated with the occurrence of an adjacent fracture, we performed a regression analysis for the multifidus muscle parameters that was adjusted for corticosteroid medication. In this adjusted analysis, still, multifidus area and multifidus fatty infiltration were shown to have no significant effect on the occurrence of adjacent vertebral fractures within one year after the index fracture (Odds Ratios 0.000, 95% CI − 0.001 to 0.001 and 0.003, 95% CI − 0.091 to 0.098, Table 2).

**Table 2:** Multiple regression analysis—Impact of multifidus area and fatty infiltration on the occurrence of adjacent vertebral fractures

	Unadjusted		Adjusted	
	OR	95% CI	OR	95% CI
Multifidus area	0.000	− 0.001, 0.000	0.000	− 0.001, 0.001
Multifidus fatty infiltration	0.013	− 0.059, 0.084	0.003	− 0.091, 0.098

*OR* odds ratio, *CI* confidence interval

## Discussion

This study aimed to evaluate the potential influence of multifidus atrophy and fatty degeneration on the incidence of adjacent vertebral compression fractures within one year after the index fracture. It was our hypothesis that an impaired multifidus muscle support increases the likelihood for an adjacent fracture.

In this retrospective analysis of 191 patients and a follow-up of one year, the only risk factor that was found to be associated with an increased risk for an adjacent vertebral fracture was a pre-existing medication with corticosteroids. Multifidus area and multifidus fatty infiltration seemed not to have a significant effect on the occurrence of such adjacent fractures.

Adjacent fractures within the first year were observed in 12% of the cases, this is a little less than what has been published in the literature where adjacent fracture rates range from 16 to 24% [4, 18, 19]. Only one prospective randomized controlled trial on adjacent fractures after kyphoplasty versus non-operative treatment reported a lower rate of 4% [20]. A reason for the slightly lower rate of adjacent fractures in the present study might be that in the authors’ institution the threshold for an operative intervention with additional posterior instrumentation in order to restore sagittal balance is low [2]. In addition, patients with OVCFs receive calcium and vitamin D and further osteological assessment and treatment is initiated as a standard. In contrast, most of the cited studies report on cohorts from more than 10 years ago when the adherence to the osteoporosis guidelines was poor [21].

Other than pre-existing medication with corticosteroids, the present study did not find specific risk factors predictive of adjacent vertebral fractures. The study showed no association between subsequent adjacent fractures and age, gender, ASA, OF fracture pattern, or medication with vitamin D or bisphosphonates. These findings are consistent with previous studies that could not find a correlation of adjacent vertebral compression fractures with age, gender, comorbidities, number of fractured levels or fracture location [4, 20, 22].

While one would expect that there was an association between HU as a measure of bone density and the occurrence of secondary adjacent fractures, this was not observed in the presented cohort. Again, this is in line with published data from several studies that examined bone quality as a risk factor for adjacent fractures [4, 23–26].

Multifidus atrophy or fatty degeneration alone does not seem to be a risk factor for adjacent level OVCFs. This is consistent with a recent study that found no differences between patients with and without osteoporotic vertebral fractures when comparing the cross-sectional areas of psoas and erector spinae muscles [27]—even though it is known that paravertebral muscles deteriorate during the first three months after the fracture [28, 29].

Multifidus cross-sectional areas (CSA) reported in the literature range from 280 mm^2^ at the level of L2 to 800 mm^2^ at the level of L5 [30].For the present study, the CSA was measured at the level of T12 and in a population of elderly and frail patients. This would explain the relatively small CSAs of about 270 mm^2^ in this study.

The limitations of this study are inherent in its retrospective study design. This includes the possibility that patients with symptomatic adjacent fractures did not present to our department. However, the authors’ institution is the only local tertiary center for spine surgery and it is uncommon that patients with subsequent fractures to the spine are referred to other centers far away.

By adjusting the regression analysis for corticosteroid medication as the only factor associated to an increased risk for adjacent fractures the study accounted for a potential selection-bias.

The cross-sectional area measurements in this study were all done at the level of T12 and, hence, have to be interpreted as a global measure of multifidus sarcopenia. It could be that in fractures of the upper thoracic spine or the lower lumbar spine, the local multifidus muscles’ condition differed from what was assessed at the level of T12.

As all participants already had at least one osteoporotic fracture suggesting a higher degree of frailty, the multifidus muscle area may be prone to intra-individual variability in this cohort. Using a larger muscle like the psoas muscle might have had the advantage of more representative measures in terms of general sarcopenia. However, it was our hypothesis that specifically a lack of multifidus muscle support destabilizes the core movements and by this increases the likelihood for subsequent vertebral fractures.

The present study did not account for potential changes in sagittal balance secondary to the degenerative changes of the multifidus muscles, as whole spine radiographs were not available for most of the patients. It has been shown that the combination of vertebral kyphosis and paraspinal muscle fatty degeneration associated with osteoporotic fractures leads to changes of the static sagittal balance [31]. This may lead to an increased risk for OVCFs in general. In addition, changes of dynamic posture control due to altered paraspinal muscle recruitment have been shown in patients with OVCFs [32]. It is important to understand that the cross-section area of the multifidus muscles as measured in this study does not necessarily resemble paraspinal muscle recruitment. It remains unclear whether changes in neuromuscular responses of the trunk are a risk factor for falls that results in a fracture or whether they are an adaptive strategy as response to pain and kyphosis.

Neuromuscular training has been shown to reduce pain and restore function in patients with low back pain [33]. Future research may investigate the correlation of core muscle function and adjacent OVCFs with electrophysiological methods and a focus on muscle recruitment and neuromuscular response.

## Conclusion

Multifidus area and multifidus fatty infiltration had no significant effect on the occurrence of adjacent vertebral fractures within one year after the index fracture. Patients with a pre-existing medication with corticosteroids were more likely to sustain an adjacent fracture.

## Data Availability

Data are available from the corresponding author on request.

## References

[1] Matsumoto T, Hagino H, Shiraki M, Fukunaga M, Nakano T, Takaoka K, Morii H, Ohashi Y, Nakamura T (2009). Effect of daily oral minodronate on vertebral fractures in Japanese postmenopausal women with established osteoporosis: a randomized placebo-controlled double-blind study. Osteoporos Int.

[2] Spiegl UJ, Anemüller C, Jarvers J-S, von der Höh N, Josten C, Heyde C-E (2019). Hybrid stabilization of unstable osteoporotic thoracolumbar vertebral body fractures: clinical and radiological outcome after a mean of 4 years. Eur Spine J.

[3] Rho Y-J, Choe WJ, Chun YI (2012). Risk factors predicting the new symptomatic vertebral compression fractures after percutaneous vertebroplasty or kyphoplasty. Eur Spine J.

[4] Teuber H, Tiziani S, Halvachizadeh S, Frey D, Sprengel K, Pape H-C, Osterhoff G (2018). Single-level vertebral kyphoplasty is not associated with an increased risk of symptomatic secondary adjacent osteoporotic vertebral compression fractures: a matched case-control analysis. Arch Osteoporos.

[5] Ning L, Wan S, Liu C, Huang Z, Cai H, Fan S (2015). New levels of vertebral compression fractures after percutaneous kyphoplasty: retrospective analysis of styles and risk factors. Pain Physician.

[6] Wang Y-T, Wu X-T, Chen H, Wang C, Mao Z-B (2014). Adjacent-level symptomatic fracture after percutaneous vertebral augmentation of osteoporotic vertebral compression fracture: a retrospective analysis. J Orthop Sci.

[7] Hida T, Shimokata H, Sakai Y, Ito S, Matsui Y, Takemura M, Kasai T, Ishiguro N, Harada A (2016). Sarcopenia and sarcopenic leg as potential risk factors for acute osteoporotic vertebral fracture among older women. Eur Spine J.

[8] Jeon I, Kim SW, Yu D (2021). Paraspinal muscle fatty degeneration as a predictor of progressive vertebral collapse in osteoporotic vertebral compression fractures. Spine J.

[9] Zhang S-B, Chen H, Xu H-W, Yi Y-Y, Wang S-J, Wu D-S (2021). Association between handgrip strength and subsequent vertebral-fracture risk following percutaneous vertebral augmentation. J Bone Miner Metab.

[10] Werner CM, Osterhoff G, Schlickeiser J, Jenni R, Wanner GA, Ossendorf C, Simmen HP (2013). Vertebral body stenting versus kyphoplasty for the treatment of osteoporotic vertebral compression fractures: a randomized trial. J Bone Joint Surg.

[11] Vaccaro AR, Oner C, Kepler CK, Dvorak M, Schnake K, Bellabarba C, Reinhold M, Aarabi B, Kandziora F, Chapman J, Shanmuganathan R, Fehlings M, Vialle L (2013). AOSpine thoracolumbar spine injury classification system: fracture description, neurological status, and key modifiers. Spine.

[12] Schnake KJ, Blattert TR, Hahn P, Franck A, Hartmann F, Ullrich B, Verheyden A, Mörk S, Zimmermann V, Gonschorek O, Müller M, Katscher S, Saman AE, Pajenda G, Morrison R, Schinkel C, Piltz S, Partenheimer A, Müller CW, Gercek E, Scherer M, Bouzraki N, Kandziora F (2018). Classification of osteoporotic thoracolumbar spine fractures: recommendations of the spine section of the german society for orthopaedics and trauma (DGOU). Global Spine J.

[13] Schneider CA, Rasband WS, Eliceiri KW (2012). NIH Image to ImageJ: 25 years of image analysis. Nat Methods.

[14] Farshad M, Gerber C, Farshad-Amacker NA, Dietrich TJ, Laufer-Molnar V, Min K (2014). Asymmetry of the multifidus muscle in lumbar radicular nerve compression. Skeletal Radiol.

[15] Goutallier D, Postel JM, Bernageau J, Lavau L, Voisin MC (1994). Fatty muscle degeneration in cuff ruptures. Pre- and postoperative evaluation by CT scan. Clin Orthop Relat Res.

[16] Scheyerer MJ, Ullrich B, Osterhoff G, Spiegl UA, Schnake KJ (2019). „Hounsfield units“ als Maß für die Knochendichte—Anwendungsmöglichkeiten in der Wirbelsäulenchirurgie (Hounsfield units as a measure of bone density-applications in spine surgery). Unfallchirurg.

[17] Soper DS (2021) A-priori sample size calculator for multiple regression. https://www.danielsoper.com/statcalc. Accessed 16 Sept 2021

[18] Mudano AS, Bian J, Cope JU, Curtis JR, Gross TP, Allison JJ, Kim Y, Briggs D, Melton ME, Xi J, Saag KG (2009). Vertebroplasty and kyphoplasty are associated with an increased risk of secondary vertebral compression fractures: a population-based cohort study. Osteoporos Int.

[19] Wardlaw D, Cummings SR, van Meirhaeghe J, Bastian L, Tillman JB, Ranstam J, Eastell R, Shabe P, Talmadge K, Boonen S (2009). Efficacy and safety of balloon kyphoplasty compared with non-surgical care for vertebral compression fracture (FREE): a randomised controlled trial. Lancet.

[20] Yi X, Lu H, Tian F, Wang Y, Li C, Liu H, Liu X, Li H (2014). Recompression in new levels after percutaneous vertebroplasty and kyphoplasty compared with conservative treatment. Arch Orthop Trauma Surg.

[21] García-Sempere A, Hurtado I, Sanfélix-Genovés J, Rodríguez-Bernal CL, Gil Orozco R, Peiró S, Sanfélix-Gimeno G (2017). Primary and secondary non-adherence to osteoporotic medications after hip fracture in Spain: the PREV2FO population-based retrospective cohort study. Sci Rep.

[22] Deibert CP, Gandhoke GS, Paschel EE, Gerszten PC (2016). A Longitudinal cohort investigation of the development of symptomatic adjacent level compression fractures following balloon-assisted kyphoplasty in a series of 726 patients. Pain Physician.

[23] Klazen CAH, Lohle PNM, de Vries J, Jansen FH, Tielbeek AV, Blonk MC, Venmans A, van Rooij WJJ, Schoemaker MC, Juttmann JR, Lo TH, Verhaar HJJ, van der Graaf Y, van Everdingen KJ, Muller AF, Elgersma OEH, Halkema DR, Fransen H, Janssens X, Buskens E, Mali WPTM (2010). Vertebroplasty versus conservative treatment in acute osteoporotic vertebral compression fractures (vertos II): an open-label randomised trial. The Lancet.

[24] Diamond TH, Champion B, Clark WA (2003). Management of acute osteoporotic vertebral fractures: a nonrandomized trial comparing percutaneous vertebroplasty with conservative therapy. Am J Med.

[25] Wang H-K, Lu K, Liang C-L, Weng H-C, Wang K-W, Tsai Y-D, Hsieh C-H, Liliang P-C (2010). Comparing clinical outcomes following percutaneous vertebroplasty with conservative therapy for acute osteoporotic vertebral compression fractures. Pain Med.

[26] Zhang H, Xu C, Zhang T, Gao Z (2017). Does percutaneous vertebroplasty or balloon kyphoplasty for osteoporotic vertebral compression fractures increase the incidence of new vertebral fractures? A meta-analysis. Pain Physician.

[27] Sollmann N, Franz D, Burian E, Löffler MT, Probst M, Gersing A, Schwaiger B, Pfeiffer D, Kirschke JS, Baum T, Riederer I (2020). Assessment of paraspinal muscle characteristics, lumbar BMD, and their associations in routine multi-detector CT of patients with and without osteoporotic vertebral fractures. Eur J Radiol.

[28] Katsu M, Ohba T, Ebata S, Haro H (2018). Comparative study of the paraspinal muscles after OVF between the insufficient union and sufficient union using MRI. BMC Musculoskelet Disord.

[29] Takahashi S, Hoshino M, Takayama K, Sasaoka R, Tsujio T, Yasuda H, Kanematsu F, Kono H, Toyoda H, Ohyama S, Hori Y, Nakamura H (2020). The natural course of the paravertebral muscles after the onset of osteoporotic vertebral fracture. Osteoporos Int.

[30] Kalichman L, Carmeli E, Been E (2017). The association between imaging parameters of the paraspinal muscles, spinal degeneration, and low back pain. Biomed Res Int.

[31] Li Q, Sun J, Cui X, Jiang Z, Li T (2017). Analysis of correlation between degeneration of lower lumbar paraspinal muscles and spinopelvic alignment in patients with osteoporotic vertebral compression fracture. BMR.

[32] Briggs AM, Greig AM, Bennell KL, Hodges PW (2007). Paraspinal muscle control in people with osteoporotic vertebral fracture. Eur Spine J.

[33] O'Sullivan PB, Phyty GDM, Twomey LT, Allison GT (1997). Evaluation of specific stabilizing exercise in the treatment of chronic low back pain with radiologic diagnosis of spondylolysis or spondylolisthesis. Spine.

